# Inhalation characteristics of asthma patients, COPD patients and healthy volunteers with the Spiromax® and Turbuhaler® devices: a randomised, cross-over study

**DOI:** 10.1186/s12890-015-0043-x

**Published:** 2015-05-01

**Authors:** Wahida Azouz, Philip Chetcuti, Harold Hosker, Dinesh Saralaya, Henry Chrystyn

**Affiliations:** Division of Pharmacy, School of Applied Sciences, University of Huddersfield, Huddersfield, UK; Paediatrics, Leeds General Infirmary, Leeds, UK; Department of Respiratory, Airedale General Hospital, Steeton, Bradford, UK; Department of Respiratory, Bradford Royal Infirmary, Bradford, UK; Inhalation Consultancy Ltd Tarn House (Formerly Division of Pharmacy, School of Applied Sciences, University of Huddersfield), 55 High Street, Yeadon, Leeds UK

**Keywords:** Adolescent, Asthma, Child, Chronic obstructive pulmonary disease, Inhalation therapy, Inhalation manoeuvre characteristics, Spiromax, Training activities, Turbuhaler

## Abstract

**Background:**

Spiromax® is a novel dry-powder inhaler containing formulations of budesonide plus formoterol (BF). The device is intended to provide dose equivalence with enhanced user-friendliness compared to BF Turbuhaler® in asthma and chronic obstructive pulmonary disease (COPD). The present study was performed to compare inhalation parameters with empty versions of the two devices, and to investigate the effects of enhanced training designed to encourage faster inhalation.

**Methods:**

This randomised, open-label, cross-over study included children with asthma (n = 23), adolescents with asthma (n = 27), adults with asthma (n = 50), adults with COPD (n = 50) and healthy adult volunteers (n = 50). Inhalation manoeuvres were recorded with each device after training with the patient information leaflet (PIL) and after enhanced training using an In-Check Dial device.

**Results:**

After PIL training, peak inspiratory flow (PIF), maximum change in pressure (∆P) and the inhalation volume (IV) were significantly higher with Spiromax than with the Turbuhaler device (p values were at least <0.05 in all patient groups). After enhanced training, numerically or significantly higher values for PIF, ∆P, IV and acceleration remained with Spiromax versus Turbuhaler, except for ∆P in COPD patients. After PIL training, one adult asthma patient and one COPD patient inhaled <30 L/min through the Spiromax compared to one adult asthma patient and five COPD patients with the Turbuhaler. All patients achieved PIF values of at least 30 L/min after enhanced training.

**Conclusions:**

The two inhalers have similar resistance so inhalation flows and pressure changes would be expected to be similar. The higher flow-related values noted for Spiromax versus Turbuhaler after PIL training suggest that Spiromax might have human factor advantages in real-world use. After enhanced training, the flow-related differences between devices persisted; increased flow rates were achieved with both devices, and all patients achieved the minimal flow required for adequate drug delivery. Enhanced training could be useful, especially in COPD patients.

## Background

Most patients with asthma or chronic obstructive pulmonary disease (COPD) require drug treatment with inhalation the major route of administration. The majority of asthma and COPD patients use their pressurised metered-dose inhaler (pMDI) incorrectly [1,2]. Major reasons for this are failure by patients to co-ordinate actuation with inhalation and failure to use a slow and deep inhalation [1,3,4]. Dry powder inhalers (DPIs) were developed with the intention of preventing errors in the co-ordination of actuation and inhalation. With a DPI, the act of inhalation de-aggregates (‘breaks up’) and releases the metered dose of drug, thereby removing the need for a patient to coordinate actuation with inhalation.

A potential drawback of DPIs is their dependency upon the patient’s inspiratory effort for delivering the correct dose of drug to the lungs [5,6]. Drug dose, particle size distribution and, ultimately, clinical effectiveness are dependent not only on peak inspiratory flow (PIF), but also acceleration rate (ACC) and inhalation time (Ti) [7-9]. This is related to the fact that drug particles are de-aggregated (a process dependent on airflow through the device [5]) before emission from the device to ensure they are small enough to reach the site of action in the small airways.

The ERS/ISAM task force has recommended that the inhalation manoeuvre when using a DPI should be forceful from the beginning and that inhalation should be continued for as long as is comfortable [10]. Disease severity may affect a patient’s ability to perform an inhalation manoeuvre with sufficient force to de-aggregate the dose, potentially jeopardising the effectiveness of inhaled medication [1,11]. Differences between devices are apparent regarding the inhalation rates that patients can achieve, which is controlled by the internal resistance to airflow inside the inhalation channel of the device [12]. This may alter the effectiveness of treatment that a patient can obtain. However, patient counselling has been shown to increase the proportion of patients achieving adequate inhalation flow rates [13,14]. Moreover, results from studies of the Turbuhaler® DPI have shown that most patients are able to inhale using flow rates necessary for effective treatment [15-17].

**Figure 1:**
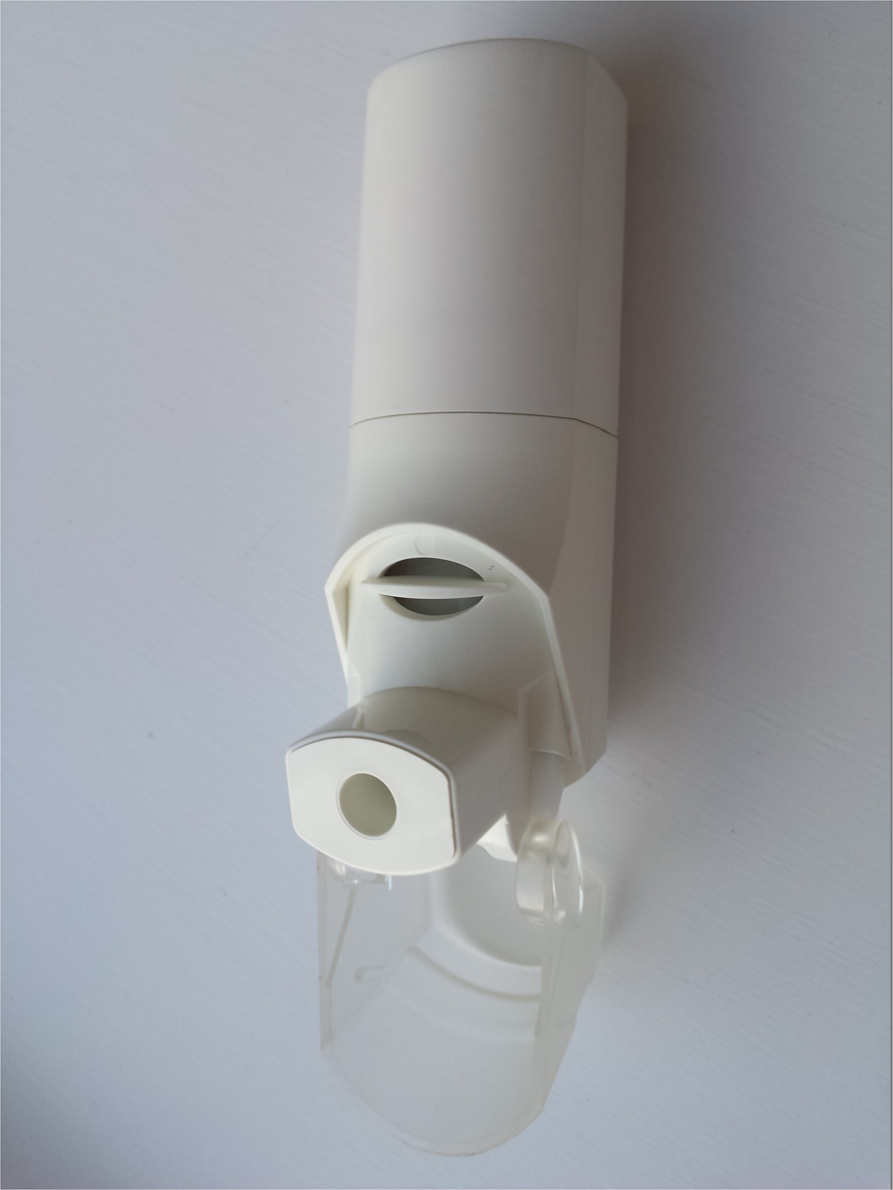
Spiromax device. Copyright of Teva UK Limited. Reproduced with permission.

The Spiromax® device (Figure 1) is a novel DPI. DuoResp® Spiromax (budesonide plus formoterol [BF] Spiromax) is approved for use in the European Union for treatment of adults (≥18 years old) with asthma and for patients with COPD for whom an inhaled corticosteroid/long-acting β_2_ agonist (ICS/LABA) combination is indicated [18]. The formulations of BF in BF Spiromax provide comparable quality and are equivalent to BF (Symbicort®) Turbuhaler at equivalent strengths [18]. Regulatory approval of Spiromax was dependent on demonstration of equivalence as opposed to superiority versus Turbuhaler, with respect to delivered dose and pharmacokinetics/pharmacodynamics.

The present study was performed to investigate PIF and related inhalation parameters of patients with stable asthma, patients with stable COPD and healthy adult volunteers when using empty Spiromax and empty Turbuhaler devices [19-22]. The effect of enhanced training on inhalation parameters was also assessed.

## Methods

This was a randomised, open-label, cross-over study involving five groups of participants: children with asthma, adolescents with asthma, adults with asthma, adults with COPD and healthy adult volunteers. The study was conducted at four centres in the United Kingdom, with recruitment from 1 November 2010 until 2 March 2011. Local research ethics committee approval was obtained (Bradford Research Ethics Committee 09/H1302/64), in addition to Research and Development approval from each participating centre. The study was conducted in accordance with good clinical practice and the declaration of Helsinki. All study participants, and parents/guardians of participants aged ≤17 years, provided signed informed consent.

### Inclusion/exclusion criteria

One hundred asthma patients were recruited as follows: children (age range 6–11 years, n = 23); adolescents (age range 12–17 years, n = 27); adults (age range 18–45 years, n = 50). Inclusion criteria for these patients were: stable asthma with no other respiratory conditions, and use of inhaled asthma medication for ≥4 weeks before study enrolment. Patients with an asthma exacerbation or who required oral prednisolone therapy during the 4 weeks preceding enrolment were excluded. Adult COPD patients (age >50 years, n = 50) were recruited, provided they had been taking inhaled COPD medication for ≥4 weeks before study enrolment. Exclusion criteria for COPD patients were asthma or other clinically relevant pulmonary disease, and an exacerbation of COPD or oral prednisolone therapy during the 4 weeks before enrolment.

### Study design, PIL training and enhanced training

**Figure 2:**
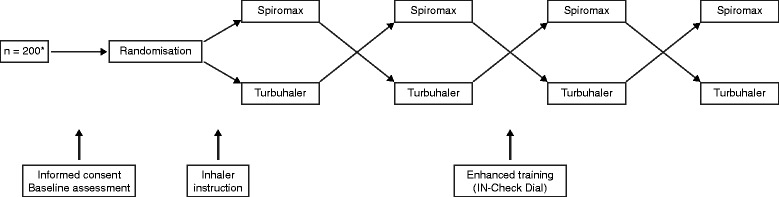
Study design.

Participants completed the study during a single clinic visit (Figure 2). Demographic data were recorded and lung function (peak expiratory flow rate [PEFR], forced expiratory volume in 1 second [FEV_1_]) was assessed by spirometry. Disease status was assessed in patients with asthma or COPD using the Asthma Control Questionnaire (ACQ; six domains, each with a scale from 0 [minimal impairment] [23] to 6 [severe impairment]) or Baseline Dyspnoea Index (BDI; three categories, each with a symptom severity scale from grade 1 [minimal impairment] to grade 5 [severe impairment]), respectively.

Both the Spiromax (Teva Pharmaceuticals) and the Turbuhaler (AstraZeneca) were provided by the manufacturers as empty devices, containing neither active drug nor powder vehicle but otherwise unmodified.

Randomisation was performed to determine which of the two devices would be used first. Training to use each DPI was provided verbally by a highly trained researcher, with instructions as per the patient information leaflet (obtained from the manufacturers of both devices). Two consecutive inhalation manoeuvres were then performed with each device.

Study participants subsequently underwent enhanced training using an In-Check Dial™ (Clement Clarke International) [5] with the device set to the resistance of a Turbuhaler. This training was provided by a highly trained researcher, who also made the inhalation manoeuvre measurements. Participants were shown their PIF and encouraged to improve it by inhaling more quickly, particularly from the start of their inhalation. Inhalation parameters for two manoeuvres performed using the faster inhalation technique were then measured in the same way as before enhanced training.

### Measurement of inhalation characteristics

A probe (ensuring an airtight seal) was placed into the inhalation channel of each inhaler distal from the opening of the mouthpiece. The probe was connected to PR3202 low differential pressure sensors (Applied Measurements Ltd, Reading, UK). The resistance of the DPI was measured before and after the insertion of the probe to ensure no changes and that an airtight seal was present. During each inhalation, the change in pressure (in mbar) with time (in milliseconds) that occurred in the inhalation channel of the device, was downloaded into an EXCEL spreadsheet.

The pressure changes were converted to inhalation flow as recommended by Clark and Hollingworth [6]. From the pressure-time readings and the corresponding inhalation flow readings the following parameters were obtained: PIF (in L min^-1^), the time to PIF (T_max_), the maximum pressure change that occurred inside the DPI (∆P; in kPa), the initial acceleration of the inhalation flow (ACCEL; in kPa sec^-1^), the inhalation volume (IV; in litres), and the duration of the inhalation (Ti; in seconds). The internal resistance of each device was measured using the technique of Clark and Hollingworth.

### Statistical analysis

For each pair of manoeuvres, the profile with the highest PIF was selected for analysis. Descriptive statistics were calculated for each parameter, and results are presented as mean and standard deviation. The percentage improvement in each inhalation parameter following training was calculated for each subject; the mean percentage improvement and standard deviation are presented.

The paired *t*-test was used to determine whether there were statistically significant differences between the Spiromax and Turbuhaler devices, both pre- and post-training. The paired *t*-test was also used to examine whether differences between values post- and pre-training were statistically significant. The statistical analysis was performed using SPSS version 17/18.

## Results

### Study participants

Demographics and baseline characteristics of the study participants are shown in Table 1. None of these individuals withdrew prematurely before completing the study. The mean ACQ score was 1.62 (standard deviation, SD, 0.95) for children with asthma (aged 6–11 years), 1.66 (0.97) for adolescents with asthma and 1.85 (0.90) for adults with asthma. Seven percent of all of the asthma patients had well controlled disease (ACQ score <0.7), 48% had partly controlled asthma (ACQ score 0.7–1.5) and 45% had poorly controlled disease (ACQ score >1.5). The majority of COPD patients had BDI grade 3 (18 subjects, 36%) or grade 4 (16 subjects, 32%); the remainder had grade 2 (n = 8) or grade 5 (n = 8).

**Table 1:** **Summary of baseline characteristics and demographic data**

**Age (yrs)**	**Height (cm)**	**Weight (kg)**	**Sex (F/M)**	**PEFR (L/min)**	**FEV**_**1**_ **(% predicted)**
**Mean (SD)**	**Mean (SD)**	**Mean (SD)**	**N (%)**	**Mean (SD)**	**Mean % (SD)**
**Children with asthma (age 6–11; n = 23)**					
8.57 (2.00)	134.26 (18.25)	37.08 (13.64)	F 9 (39.13)	182.74 (88.01)	Not applicable
			M 14 (60.87)		
**Adolescents with asthma (age 12–17; n = 27)**					
14.52 (1.55)	160.54 (7.63)	57.73 (12.17)	F 14 (51.85)	310.07 (104.36)	64.63 (15.89)
			M 13 (48.15)		
**Adults with asthma (age 18–45; n = 50)**					
34.74 (7.69)	168.06 (4.92)	75.48 (10.49)	F 29 (58.00)	329.48 (101.51)	69.28 (16.63)
			M 21 (42.00)		
**Adults with COPD (age > 50; n = 50)**					
66.82 (7.98)	168.74 (6.94)	78.09 (13.62)	F 28 (56.00)	216.48 (93.25)	51.88 (21.90)
			M 22 (44.00)		
**Healthy volunteers (age 18–45; n = 50)**					
32.62 (7.34)	171.20 (7.86)	73.82 (14.07)	F 29 (58.00)	479.30 (127.58)	95.76 (14.31)
			M 21 (42.00)		

### Asthma or COPD medication use reported at start of study

Salbutamol was taken by >90% of the patients with asthma and by 82% of those with COPD. Percentages of salbutamol recipients receiving the drug via an MDI (with or without a spacer) were as follows: 100% of the children with asthma, 85%; of the adolescents with asthma, 80%; of the adults with asthma, and 60% of the 80%; COPD patients.

Other medications used by asthma patients were Seretide™ Accuhaler™ (34.8-52%) and Symbicort Turbuhaler (30.4-51.9%). Of the COPD patients, 74% were prescribed salbutamol.

### Device characteristics

The internal resistance of the empty Spiromax device was 0.100 (cmH20)½ (l/min)^-1^ (equivalent to 0.0313 kPa½ (l/min)^-1^), which is similar to the resistance of the commercially available Spiromax device. The internal resistance of the Turbuhaler device used was 0.107 (cmH20)½ (l/min)^-1^ (equivalent to 0.0355 kPa½ (l/min)^-1^) and this is similar to commercially available Symbicort® Turbuhaler [24].

### Inhalation parameters after standard PIL training

**Figure 3:**
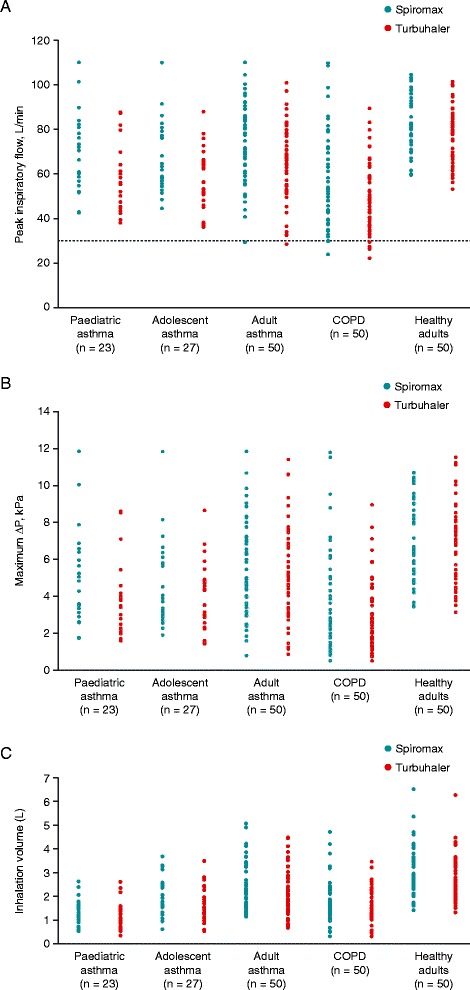
Individual peak inspiratory flow rates **(A)**, maximum pressure change (ΔP) **(B)** and inhalation volume **(C)** before enhanced training. In graph **(A)**, the horizontal dotted line represents 30 L/min (minimal flow for adequate drug delivery).

PIF, maximum change in pressure (∆P) and inhalation volume (IV) were significantly higher with Spiromax than with the Turbuhaler device (Table 2). Differences between the two inhalers in PIF were highly significant in all five study groups (p ≤ 0.0001), while statistical significance (p < 0.05) was observed with maximum ∆P in the four patient groups. No statistical difference was observed for maximum ∆P in the healthy adult group for Spiromax versus Turbuhaler. Distributions of individual patient values for PIF, maximum ∆P and IV are depicted in Figure 3. Pre-training, there were trends towards slightly higher inspiratory ACC with Spiromax, with statistically significant differences in the COPD and healthy adult groups (Table 2). Figure 3a shows that post-PIL training, one adult with asthma and one COPD patient inhaled <30 L/min with Spiromax and that one adult with asthma and five patients with COPD inhaled <30 L/min with the Turbuhaler. IV was also significantly higher with Spiromax versus Turbuhaler in all study groups.

**Table 2:** **Inhalation parameters before enhanced training**

	**Children with asthma (n = 23)**	**Adolescents with asthma (n = 27)**	**Adults with asthma (n = 50)**	**Adults with COPD (n = 50)**	**Healthy adults (n = 50)**
**Spiromax**					
**PIF, L/min**	69.5^‡^	67.9^‡^	74.4^‡^	57.5^‡^	85.0^‡^
	(17.2)	(15.1)	(18.1)	(21.0)	(13.6)
**Max** Δ**P, kPa**	5.0^†^	4.7*	5.7*	3.7^†^	7.3
	(2.4)	(2.2)	(2.6)	(2.7)	(2.3)
**ACC, kPa/s**	13.6	12.1	15.6	11.0*	15.9*
	(11.8)	(8.8)	(15.7)	(12.8)	(13.5)
**Inhalation volume, L**	1.50^†^	2.03^†^	2.39^†^	1.82^†^	2.98^*^
	(0.6)	(0.81)	(1.03)	(0.88)	(1.02)
**Turbuhaler**					
**PIF, L/min**	58.5	57.8	65.4	50.1	78.0
	(14.7)	(13.4)	(17.5)	(16.2)	(11.8)
**Max** Δ**P, kPa**	3.9	3.9	5.1	3.1	7.0
	(2.0)	(1.8)	(2.6)	(2.0)	(2.1)
**ACC, kPa/s**	10.2	11.4	13.0	8.4	12.8
	(7.7)	(7.2)	(12.1)	(9.5)	(9.6)
**Inhalation volume, L**	1.25	1.68	2.13	1.58	2.80
	(0.57)	(0.74)	(1.01)	(0.69)	(0.92)

Data shown are mean (standard deviation). *p < 0.05 vs Turbuhaler; ^†^p < 0.01 vs Turbuhaler; ^‡^p ≤ 0.0001 vs Turbuhaler.

Mean time to PIF was generally similar for the two devices, ranging between 0.61 and 1.02 seconds across the five study groups with Spiromax and between 0.79 and 1.19 seconds with Turbuhaler (data not shown). The only group with a significant difference in time to PIF was COPD patients, where it was significantly shorter with Spiromax (0.68 vs 0.96 seconds, p = 0.0135). In each study group, Ti was similar with both devices; the range of mean values across the five study groups was 1.81–2.94 seconds with the Spiromax device and 1.94–3.02 seconds with Turbuhaler.

### Effects of enhanced training

Enhanced training, when compared to standard PIL training, significantly improved PIF, ACC, maximum ∆P and IV (p < 0.05) in all study groups and with both inhalers. Percentage improvements following enhanced training were slightly larger with Turbuhaler than with Spiromax (Table 3). With both devices, the parameter with the highest percentage improvements in response to enhanced training was ACC (Table 3). IV was the parameter with the smallest percentage improvements.

**Table 3:** **Inhalation parameters after enhanced training, and percentage change versus pre-training values**

	**Children with asthma (n = 23)**	**Adolescents with asthma (n = 27)**	**Adults with asthma (n = 50)**	**Adults with COPD (n = 50)**	**Healthy adults (n = 50)**
**Spiromax**					
**PIF ± SD, L/min**	77.99 ± 17.64^†^	83.87 ± 15.12^‡^	85.45 ± 14.60^‡^	68.08 ± 18.48^‡^	98.68 ± 9.25^‡^
**(Change ± SD, %)**	(14.18 ± 22.51)	(26.34 ± 22.83)	(19.31 ± 26.65)	(25.27 ± 33.36)	(18.93 ± 22.39)
**Max** Δ**P ± SD, kPa**	6.25 ± 2.64	7.11 ± 2.50*	7.36 ± 2.33^†^	3.94 ± 2.09	9.62 ± 1.66
**(Change ± SD, %)**	(35.24 ± 59.55)	(64.64 ± 60.13)	(49.30 ± 74.81)	(35.94 ± 81.15)	(46.36 ± 58.85)
**ACC ± SD, kPa/s**	19.10 ± 14.63	26.72 ± 18.42	30.02 ± 25.30*	18.79 ± 17.07*	32.21 ± 17.19
**(Change ± SD, %)**	(102.11 ± 171.24)	(189.28 ± 234.48)	(247.72 ± 482.94)	(152.83 ± 233.91)	(212.09 ± 284.44)
**Inhalation volume ± SD, L**	1.58 ± 0.60^†^	2.13 ± 0.67^†^	2.38 ± 1.12^†^	1.90 ± 0.90^†^	3.07 ± 1.05^†^
**(Change ± SD, %)**	(14.68 ± 42.03)	(10.09 ± 21.40)	(1.25 ± 26.19)	(14.73 ± 59.64)	(6.41 ± 27.99)
**Turbuhaler**					
**PIF ± SD, L/min**	69.46 ± 16.18	74.31 ± 12.94	76.73 ± 15.01	60.09 ± 16.95	90.36 ± 11.00
**(Change ± SD, %)**	(20.03 ± 17.51)	(32.68 ± 27.56)	(22.17 ± 28.32)	(24.06 ± 25.56)	(18.16 ± 21.92)
**Max** Δ**P ± SD, kPa**	5.70 ± 2.53	6.38 ± 2.21	6.86 ± 2.51	4.37 ± 2.44^§^	9.30 ± 2.09
**(Change ± SD, %)**	(50.99 ± 40.47)	(83.35 ± 82.04)	(57.12 ± 89.53)	(60.30 ± 73.15)	(44.33 ± 56.21)
**ACC ± SD, kPa/s**	19.76 ± 12.37	23.58 ± 12.52	25.96 ± 20.29	15.72 ± 13.98	30.12 ± 14.34
**(Change ± SD, %)**	(214.36 ± 295.99)	(237.34 ± 365.66)	(188.77 ± 271.47)	(254.32 ± 426.12)	(275.05 ± 389.83)
**Inhalation volume ± SD, L**	1.29 ± 0.53	1.77 ± 0.56	2.11 ± 0.90	1.66 ± 0.71	2.79 ± 0.96
**(Change ± SD, %)**	(9.58 ± 31.29)	(15.52 ± 30.85)	(5.88 ± 34.35)	(11.26 ± 40.03)	(0.69 ± 19.91)

Data shown are mean ± standard deviation *p < 0.05 vs Turbuhaler; ^†^p < 0.01 vs Turbuhaler; ^‡^p < 0.0001 vs Turbuhaler; ^§^p < 0.01 vs Spiromax.

After enhanced training, PIF remained significantly higher with Spiromax versus Turbuhaler in all study groups (p < 0.01; Table 3). Numerically or significantly higher values with Spiromax versus Turbuhaler were also observed for maximum ∆P, ACC and IV after enhanced training, with the exception of maximum ∆P in COPD patients (Table 3). Time to PIF was shorter with both devices after enhanced training, with study group mean values ranging between 0.48 and 0.56 seconds with Spiromax, and between 0.43 and 0.56 seconds with Turbuhaler. There were no significant post-training differences between the devices in time to PIF for any of the study groups. Slight reductions were apparent in Ti post-training, but mean values remained similar with both devices.

## Discussion

This study shows that most patients, regardless of age or underlying disease severity, can achieve satisfactory inhalation manoeuvre parameters through empty versions of the Spiromax and Turbuhaler dry powder inhalers. Enhanced training was useful to improve the inhalation characteristics of those patients with peak inhalation flows <30 L/min, especially COPD patients using the Turbuhaler. The increases in response to enhanced training highlight that there is room for improvement and that training patients to use these devices can be valuable. Although better inhalation characteristics were achieved when inhaling through the empty Spiromax, it is doubtful that this would translate into clinical differences between the devices since equivalence between them has been shown among highly trained patients [18]. PIF values were lower among the COPD patients and the young asthma patients than among the adults with asthma, and the healthy volunteers achieved the highest PIF; these results were as expected [12].

There were statistically significant differences in key parameters (PIF, maximum ∆P and ACC) between the Spiromax and Turbuhaler, with greater improvements overall typically seen in the Spiromax group. The exception was the higher maximum ∆P value achieved by the Turbuhaler group, limited to COPD patients after enhanced training. This result must be considered in the context that (1) after enhanced training, all COPD patients in both groups achieved the minimal flow (30 L/min) required for adequate drug delivery and (2) prior to enhanced training, one COPD patient using Spiromax, as opposed to five COPD patients using Turbuhaler, did not achieve the minimal required flow rate. It may be argued that these results are more reflective of clinical practice than the finding that no patients failed to reach the 30 L/min threshold after enhanced training. The reason for this is that few patients in clinical practice are likely to receive training that is comparative to the enhanced training of this study. Also, several studies have highlighted poor inhalation technique with DPIs in clinical practice [25,26]. Usually, differences in flow characteristics between DPIs are related, at least in part, to different airflow resistance [27]. However, the present results show the reverse. Since the patients likely used similar inspiratory effort with both devices, it would be expected that values for ∆P and PIF would be higher for Turbuhaler because of the higher resistance of this device. However these values were slightly higher for Spiromax and suggest that additional factors can influence the inhalation characteristics of an inhalation manoeuvre.

Consistent with previous studies [11-13] enhanced training produced significant improvement in the inhalation parameters of individuals using both devices. Percentage increases in response to training were generally larger with Turbuhaler than Spiromax. Comparison between the two devices of the effects of enhanced training was consistent across the study groups: asthma patients of different ages, COPD patients and healthy adults. Smaller post enhanced training improvements with the Spiromax device may reflect increased ease of use or concordance during use and so the scope for improvement is reduced if patients have good technique from the outset. This notion is strengthened by the fact that a proportion of patients in the present study were already users of the Turbuhaler device, since pre-existing expertise in using the Turbuhaler should in theory reduce the scope for improvement with this device. The greatest improvements were in the acceleration rate (with a faster time to the PIF), highlighting the importance of training patients to inhale as fast as they can from the start to ensure better de-aggregation of the dose [7]. An understanding of the time taken to device mastery (absence of critical errors) and maintenance of device mastery with Spiromax and Turbuhaler, and the identification of long-term real-life use of these two devices in a population of adults with asthma, await further study [28].

In addition to possible ‘increased ease of use’ or reduced need for training with Spiromax, patients may be more familiar with the ‘look’ of the Spiromax inhaler compared with the Turbuhaler because Spiromax has contours similar to those of an MDI (DuoResp® Spiromax PIL). The majority of patients with asthma (at least 80%) or COPD (approximately 60%) were using an MDI (for salbutamol) at the start of the study, compared with 30.4–51.9% who were using the Turbuhaler. However, whether this contributed to the significant differences seen between the devices (favouring Spiromax) is beyond the scope of the current study. Furthermore, this finding does not account for the significant differences between the devices seen in the healthy adult group. Neither patient preference nor opinion (such as familiarity) of the devices were assessed at any point during the study. The evaluation of patient device preference (Turbuhaler and Spiromax) awaits further study.

An important limitation of this study is the open-label design, with training provided by a highly trained researcher who also made the inhalation manoeuvre measurements. This may have introduced the potential for bias – there is a possibility that study participants would use a device they recognise slightly differently from a new device with which they are unfamiliar. Completion of the study at one clinic visit is another drawback in relation to applicability of the results because, in clinical practice, inhalers are used in a variety of different environments over long periods of time. It would be useful to investigate whether the improvements resulting from enhanced training would be maintained over time during routine use. It is also yet to be established how flow and pressure profiles might differ with empty devices (as used here) versus those administrating a drug dose. Study devices were otherwise unaltered, however, and resistance measurements were not affected by the absence of drug and powder vehicle. An additional limitation is the lack of information regarding drug delivery or clinical effect; given the current study design, a robust approach to clinical endpoints was not feasible, but the data suggest that comparisons involving clinical endpoints should be of interest.

## Conclusions

In conclusion, numerically or significantly higher pre-training inhalation flow-related values were noted for empty Spiromax versus empty Turbuhaler, with PIF results showing the largest differences. Airflow resistance is slightly higher with Turbuhaler than with Spiromax, although it may be considered as broadly similar in the two devices. Although resistance has a major influence on inhalation characteristics, there might be other human factors in real-world use. After enhanced training, the flow-related differences persisted, but increased flow rates were achieved with both devices to the point that the minimal flow required for adequate drug delivery was reached by all patients, including those who inhaled <30 L/min before enhanced training (Spiromax: one adult asthma patient and one COPD patient; Turbuhaler: one adult asthma patient and 5 COPD patients). These results suggest that PIL training is effective for Spiromax and Turbuhaler users, and enhanced training may benefit selected patients with impairment in generating inspiratory force. The acceleration improvements with a faster time to PIF highlight the importance of encouraging patients to inhale as fast as they can from the start of the inhalation manoeuvre.
